# Wearable optical coherence tomography angiography probe with extended depth of field

**DOI:** 10.1117/1.JBO.30.1.016003

**Published:** 2025-01-22

**Authors:** Xiaochen Li, Xiangyu Guo, Xinyue Wang, Lingqi Jiang, Mingxin Li, Xiaochuan Dai, Qun Hao, Jingjing Zhao, Yong Huang, Liqun Sun

**Affiliations:** a Tsinghua University, State Key Laboratory of Precision Measurement Technology and Instruments, Department of Precision Instrument, Beijing, China; b Tsinghua University, School of Biomedical Engineering, Beijing, China; c Beijing Institute of Technology, School of Optics and Photonics, Beijing, China; d Huazhong University of Science and Technology, Institute of Medical Equipment Science and Engineering, Wuhan, China

**Keywords:** wearable optical coherence tomography, brain imaging, diffractive optical elements, depth of field, optical coherence tomography angiography, freely moving mice, high-resolution imaging, biomedical imaging

## Abstract

**Significance:**

Optical coherence tomography (OCT) is widely utilized to investigate brain activities and disorders in anesthetized or restrained rodents. However, anesthesia can alter several physiological parameters, leading to findings that might not fully represent the true physiological state. To advance the understanding of brain function in awake and freely moving animals, the development of wearable OCT probes is crucial.

**Aim:**

We aim to address the challenge of insufficient depth of field (DOF) in wearable OCT probes for brain imaging in freely moving mice, ensuring high lateral resolution while capturing brain vasculature across varying heights.

**Approach:**

We integrated diffractive optical elements (DOEs) capable of generating beams with an extended DOF into a wearable OCT probe. This design effectively overcomes the traditional trade-off between lateral resolution and DOF, enabling the capture of detailed angiographic images in a dynamic and uncontrolled environment.

**Results:**

The enhanced wearable OCT probe achieved a lateral resolution superior to 8  μm within a 450  μm axial range. This setup allowed for high-resolution optical coherence tomography angiography (OCTA) imaging with extended DOF, making it suitable for studying brain vasculature in freely moving mice.

**Conclusions:**

The incorporation of DOEs into the wearable OCT probe represents a significant advancement in wearable biomedical imaging. This technology facilitates the acquisition of high-resolution angiographic images with an extended DOF, thus enhancing the ability to study brain function in awake and naturally behaving animals.

## 1 Introduction

The advancement of biomedical imaging technology has significantly enhanced our understanding of complex physiological processes and the diagnosis of various health issues.^1^^–^^3^ Understanding the cerebral hemodynamics in rodents is a crucial question of interest in the fields of neuroscience, physiology, psychology, and imaging.^4^^,^^5^ The vast majority of traditional imaging techniques require the subject animals to remain still or be anesthetized to ensure image quality and stability, classic optical imaging setups for mouse brains typically involve fixing the mouse’s head beneath the lens, and throughout the imaging process, gas anesthetics are delivered to the mouse through a tube and a mask. These setups are widely used in two-photon microscopy,^6^ photoacoustic imaging,^7^ and optical coherence tomography (OCT).^8^ However, under anesthesia, multiple physiological indicators can be affected, leading to research results that may not fully represent the true physiological state.^9^ Therefore, there is a growing awareness of the necessity to image the brain under fully awake conditions.

Researchers have developed various experimental setups to study the awake brain. For example, fixing the mouse’s head and placing it on a spherical treadmill with air suspension allows the mouse to move freely to release pressure.^10^ However, this kind of device has a relatively large vertical size and is not easy to use. In 2020, Li et al.^11^ used a cage with a flat-bottomed air-suspended board, fixing the mouse’s head and placing it on the board. Unlike mice on the spherical treadmill that need to keep running, mice on the flat-bottomed suspension board can choose to move or rest on their own. Many studies adopted these similar approaches,^12^^,^^13^ but all faced the problem of large volume. Therefore, the development of wearable probes capable of acquiring high-resolution images from freely moving, awake mice has emerged as a promising solution. In fact, photoacoustic imaging,^14^ two-photon microscopy,^15^ and laser speckle contrast imaging^16^ have all developed wearable probes for studying the brains of freely moving animals.

Optical coherence tomography angiography (OCTA) is the application for measuring and visualizing 3D mapping of blood perfusion, achieved by mathematically analyzing the red blood cells’ motion-induced temporal changes of scattering signals.^17^ Compared with other optical techniques, OCTA allows rapid, high-sensitivity, contrast-free imaging with micron-scale resolution, making it a promising choice for brain imaging. Inspired by the wearable idea, we developed a wearable OCTA probe capable of long-term monitoring of cerebral blood flow in mice.^18^ Experimental results have demonstrated the feasibility of high-resolution vascular imaging of the freely moving mouse brain under natural conditions.

In subsequent research, we found that the wearable probe also faces the trade-off between lateral resolution and depth of field (DOF).^19^ Specifically, achieving higher resolution requires the imaging lens with a large numerical aperture, resulting in a short DOF. When imaging the mouse brain, the presence of height variations on the brain surface makes it challenging to obtain high-resolution images of large fields of view (FOV) without stitching small fields. However, stitching introduces complexities in both hardware and software and increases data acquisition time.^20^^–^^22^ Therefore, a better solution is to extend the DOF of the beam without sacrificing resolution. In 2022, Zhao et al. from Stanford University developed a flexible method for generating long DOF needle-shaped beams (NBs) using diffractive optical elements (DOE).^23^ By placing the DOE at the back focal plane of the lens, a multi-focus optical field distribution is generated along the axial direction. By appropriately selecting the number and distribution of focal points, an NB with extended DOF is achieved. This method was later successfully applied to photoacoustic imaging and OCTA for studies on the mouse brain.^24^^,^^25^

In this study, to address the issue of insufficient DOF when facing height variations in mouse brain imaging using the wearable OCTA probe, we incorporated the aforementioned DOE capable of generating NB into our wearable probe, overcoming the constraint relationship between lateral resolution and DOF. We achieved lateral resolution superior to 8  μm within a 450  μm axial space, capable of achieving high-resolution OCTA imaging with extended DOF by the wearable probe.

## 2 Materials and Methods

### 2.1 System Overview

**Fig. 1:**
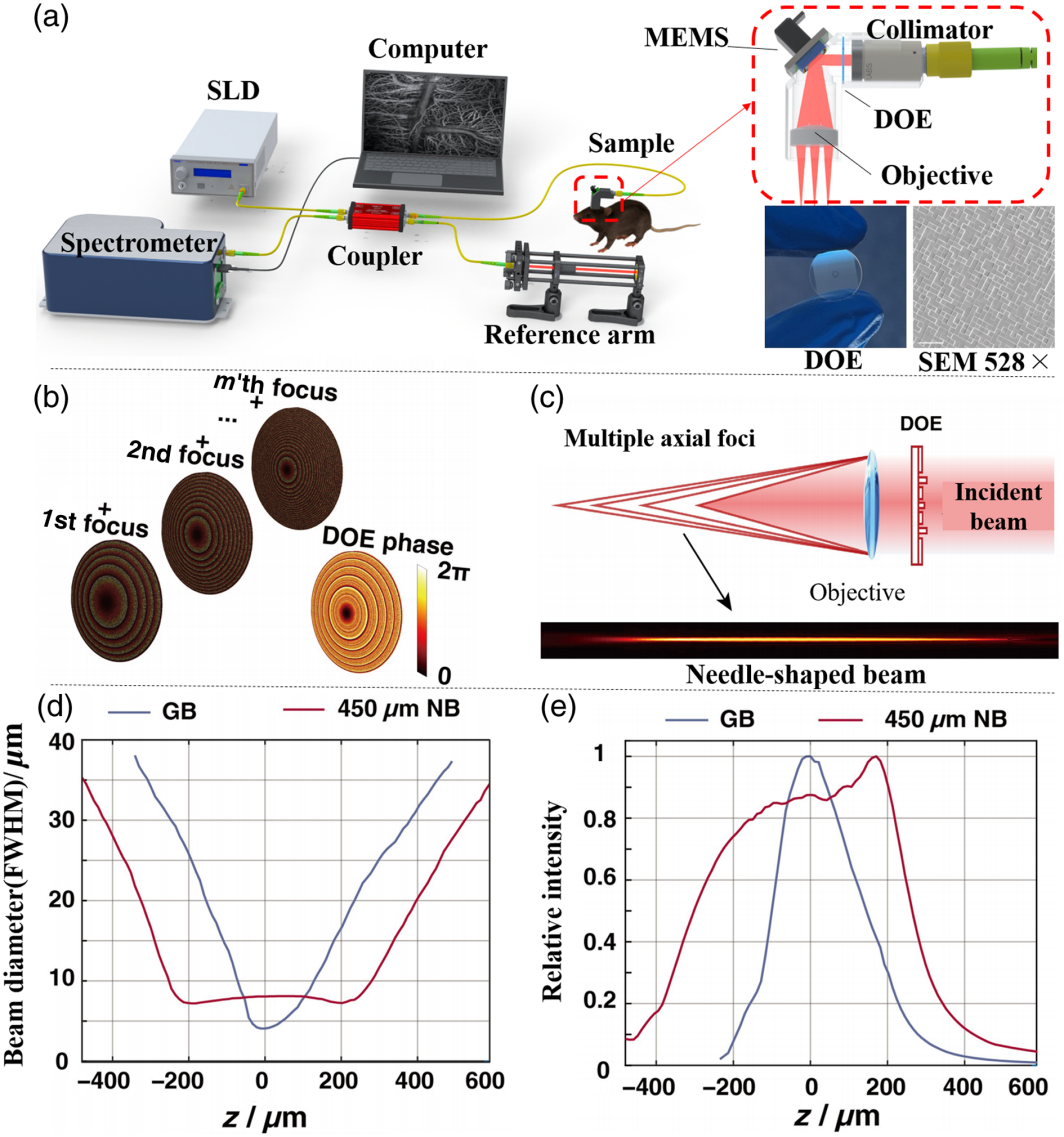
Experimental system configuration and the principle of NB. (a) Setup of the imaging system and a detailed optical configuration of the wearable OCTA probe with extended DOF. SLD: super luminescent diode; MEMS: micro-electromechanical systems. (b) DOE phase pattern for an NB composed of multiple phases to generate m foci. (c) Schematic diagram of generating an NB. (d) Diameters of the used GB and 450  μm NB along the axial position. (e) Relative intensity of the used GB and 450  μm NB along the axial position.

The wearable OCTA probe with extended DOF proposed in this study adopts the main structure of our previous wearable probe,^18^ shown in Fig. 1(a), in addition to the existing three key components (collimator, MEMS mirror, and objective lens), the probe incorporates DOE that can work with the objective lens to generate NB as described in our previous work.^24^ Figure 1 illustrates the generation of NB using a DOE. By manipulating the beam phase with DOE, multiple foci are generated along the optical axis within the vicinity of the objective lens.^23^ These foci collectively form an NB with extended DOF. Each axial focus corresponds to one phase distribution, and the final phase loaded onto the DOE is the superposition of all foci’ phase distributions, as depicted in Fig. 1(b). Through optimization of the initial phase and pixel position of each axial focus, cross-talk between phase distributions corresponding to different focal points is suppressed. This ensures uniform axial intensity of the NB generated by the DOE and suppresses the generation of sidelobes, as illustrated in Fig. 1(c).

Based on our previous finding, imaging the mouse brain with an FOV of about 3 mm requires at least 400  μm DOF.^24^ Therefore, the target DOF of the NB used in this experiment was set to 450  μm. The number of required foci is determined by the target DOF, based on the principle that the distance between adjacent focal points should be less than the Rayleigh length (RL), typically set to 0.5 to 0.8 times RL.^23^ The Rayleigh length is calculated as $\mathrm{RL}=(\lambda /n)f^{2}/(\pi r^{2})$, where r is the beam waist radius, n is the refractive index, f is the focal length, and λ is the center wavelength. In our design, we used a Gaussian beam (GB) diameter of 4.6 mm, resulting in a Rayleigh length of ∼18.47  μm. Based on this calculation, we determined that 49 focal points arranged in a 7×7 grid would be sufficient to achieve the desired 450  μm DOF, ensuring uniform intensity distribution across the depth range with appropriately spaced focal points.

After determining the number of focal points, we found that overlaying the phases of multiple foci leads to uneven axial intensity. To address this, the positions of the foci need to be optimized. We calculate the optimized axial intensity distribution using Fresnel diffraction and further adjust the spacing between adjacent foci based on the current intensity distribution. After this iterative optimization process, we obtain a beam with uniformly distributed axial intensity.

The DOE used in this study features an effective optical area of 1  cm×1  cm, containing 1000×1000  pixels with a pixel size of 10  μm. The DOE has a physical diameter of 12.7 mm, a thickness of 500  μm, and is mounted in a custom-designed slot with a 700  μm width for easy installation. The DOE was fabricated using four rounds of lithography, and after fabrication, it was cut into a circular shape with a diameter of 12.7 mm and was inserted into the pre-designed slot in the probe, as shown in Fig. 1(a).

The wearable probe with extended DOF and our homebuilt spectral domain OCT (SD-OCT) system are shown in Fig. 1(a). The light source is a broadband super luminescent diode (SLD-371HP3, Superlum, 850±25  nm, 15 mW). A 2×2 coupler (BXC45, Thorlabs, Newton, United States) was used to divide the light into the sample arm and reference arm. The reflected light from the two arms was directed to a home-built spectrometer with 0.05 nm resolution, 2048 pixels, and a maximum speed of 70 kHz.

In our customized wearable probe, the beam from the optical fiber was collimated by a collimator and then directed to a two-axis MEMS scanning mirror (A7B1.1, Mirrorcle Tech, Richmond, California) which was put on the front focal plane of an achromatic doublet lens (MAD303-B; diameter, 12.7 mm; focal length, 19 mm) for telecentric scanning. The collimator used is a fiber-coupled fixed-focus collimator (F260APC-850, Thorlabs) with a waist diameter of 3.32 mm. The MEMS scanning mirror has a reflective diameter of 3.6 mm and a maximum tilt range of ±7  deg.

The collimator, the MEMS mirror, and the objective are integrated through a customized lightweight one-piece 3D-printed mount which is specifically designed and precisely machined to simplify assembly and alignment of the components as well as removal and replacement. This one-piece 3D-printed mount enhances the stability of the probe during mouse movement and minimizes motion artifacts during imaging. The size and weight of this probe increased a little compared with the previous one due to the introduction of DOE, with dimensions of 37  mm×34  mm×15  mm, and weighs 10 g. The beam diameter and relative intensity shown in Figs. 1(d) and 1(e) were measured by a microscope system together with a beam profiler with 10  μm as the axial scanning step size (experimental setup can be found in our previous work^24^). The NB with the addition of the DOE can maintain a spot size smaller than 8  μm within an axial range of 450  μm. By contrast, although the traditional GB has a smaller spot size near the focal plane, it rapidly expands along the axial range, only able to maintain a size smaller than 8  μm within an axial range of 160  μm. Similarly, the intensity of the GB decays rapidly with axial distance, whereas the intensity of the NB remains relatively stable over a larger distance.

**Fig. 2:**
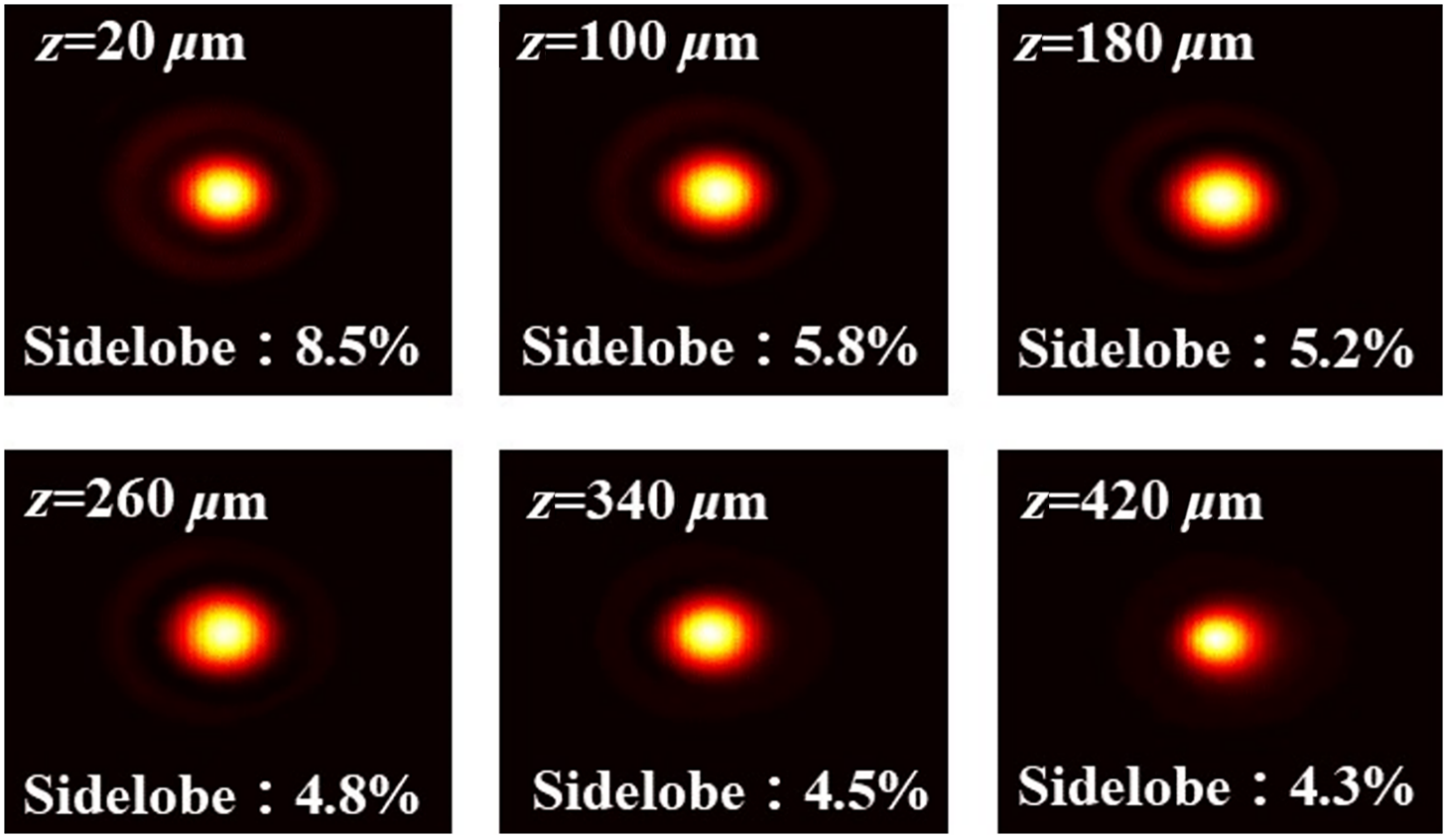
Profile of the spot (x−y) and the proportion of sidelobes at different axial positions.

Experimental measurements showed that after introducing the DOE, the power in the sample arm was ∼57% of the original power. To address the potential impact of sidelobes and insertion loss introduced by the DOE on system performance, we optimized the NB design to balance DOF extension and energy concentration. Figure 2 shows the beam profiles at different axial positions. Six positions with a step size of 80  μm were selected to demonstrate the 400  μm axial distance in the middle of the beam. It can be observed that at these axial positions, only a maximum of 8.5% of the energy is dispersed into the sidelobes near the proximal end, with less than 6% energy distributed in the sidelobes at other positions. Most of the energy is concentrated in the central main lobe. Figure 2 demonstrates that this NB has a high energy utilization rate, which is more conducive to imaging biological tissues compared with Bessel beams with a higher proportion of sidelobe energy.^26^^–^^28^

**Fig. 3:**
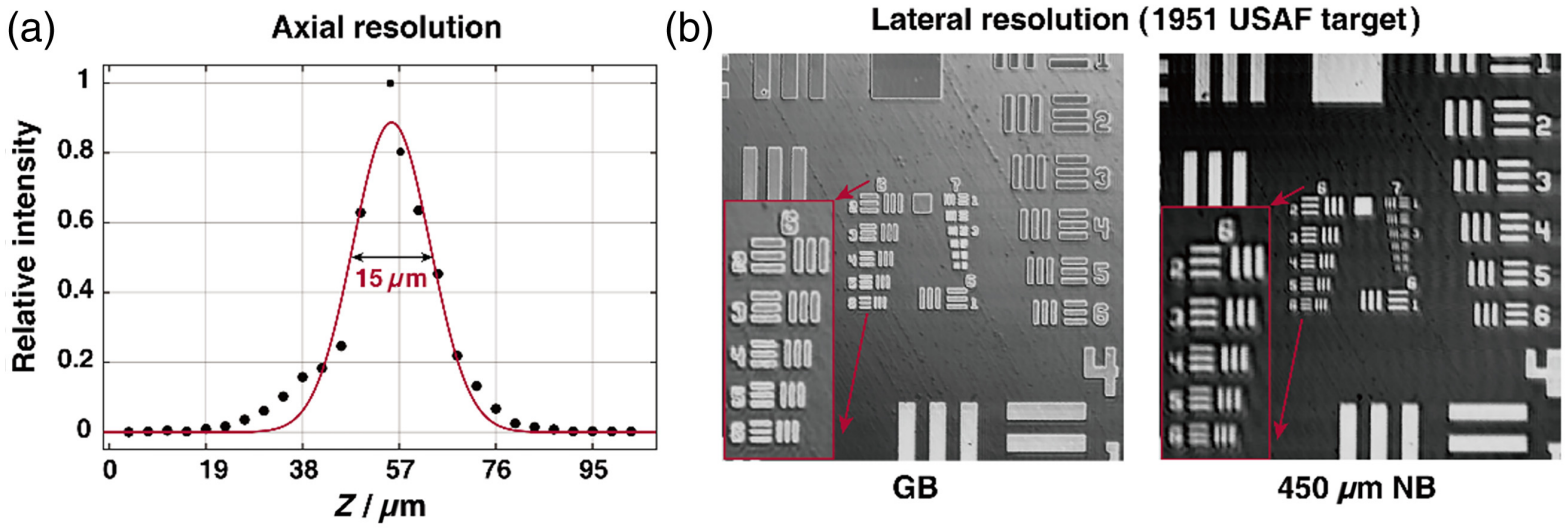
Performance of the probe. (a) Experimental estimation of the axial resolution. (b) Experimental estimation of the lateral resolution.

The axial resolution was evaluated by measuring the signal of a mirror, shown in Fig. 3, the full width at half-maximum was measured to be 15  μm. To evaluate the lateral resolution, we imaged the 1951 USAF resolution target. Before the introduction of the DOE, the smallest lines that can be resolved by GB are group 6, element 6. This corresponds to 114 line pairs (lp/mm) and a lateral resolution of ∼4.4  μm. After incorporating the DOE, the lateral resolution of the system slightly decreased compared with the resolution of the GB at the focal plane. It could resolve the 6th group, 5th element, corresponding to 102  lp/mm, and a lateral resolution of ∼4.9  μm. The NB design maintains a relatively stable lateral resolution over the entire axial depth range. Although there is a slight decrease in lateral resolution below 4.9  μm at deeper locations, the lateral resolution remains within acceptable limits with variations remaining below 8  μm over the entire 450  μm depth range.

As demonstrated in Fig. 1(b) of our previous work,^24^ the NB exhibits a significant advantage in terms of extended DOF compared with the GB. The x−z profile and cross-sectional views of the beams reveal that while the NB maintains stable lateral resolution over the extended DOF, the GB, although it offers higher resolution within a shorter DOF, experiences a rapid degradation in resolution as the depth increases. Consequently, although the NB achieves a higher resolution within the extended DOF range, its lateral resolution is slightly reduced compared with the GB at the focal plane. This advantage is particularly beneficial for imaging biological samples with non-flat surfaces, such as the mouse brain, in large FOV imaging.

### 2.2 Animal Preparation

All procedures involving mice were approved by the Institutional Animal Care and Use Committee of Tsinghua University. Wild-type mice (C57BL/6, 9 weeks to 3 months old; weight, 20 to 30 g) were used in all the experiments.

The craniotomy surgery was performed in the following manner: Initially, mice were anesthetized with isoflurane (3% for induction, 1.5% during surgery). Subsequently, a toe pinch test was carried out to ensure the depth of anesthesia. After confirming the mouse’s proper anesthesia, it was carefully positioned on a stereotactic frame while maintaining its body temperature with a heating pad. Before commencing the surgery, the ophthalmic ointment was applied topically to safeguard the corneal surfaces from drying and potential damage due to extended exposure to the illuminating light source. A scalp incision was made, followed by conducting a craniotomy using a drill.^29^ Once the skull was carefully removed, the brain was covered with 2% agarose and then a cover glass (with a thickness of 0.17 mm and a diameter of 6 mm) was affixed to the skull to minimize any motion of the exposed brain. In addition, a custom aluminum head post was securely attached to the skull using dental cement.

### 2.3 Data Acquisition and Processing

In this work, we employed a stepwise raster scanning procedure to collect volumetric data. The slow scanner (y) was driven by a step waveform with a total of 800 steps. At each step, five consecutive B-scans were acquired to analyze dynamic flow signals. Each of these B-scans consisted of 1024 A-lines in the fast-scanning direction. Adjacent B-scans were paired for blood flow extraction at each scanning step y. The original spectral interference fringe signal S (k,x,t) underwent a Fourier transformation along the wavenumber direction, resulting in the backscattered profile $C_{\mathrm{OCT}}$ (z,x,t) in the depth domain. This profile included both amplitude I and phase Φ information:

$$C_{\mathrm{OCT}}(z,x,t)=I(z,x,t)e^{-i\Phi (z,x,t)}.$$ (1)

The cross-sectional angiogram was then created by subtracting the amplitude component I (z,x,t) between paired B-scans:

$${\mathrm{OCTA}=|I(z,x,t}_{i}{)-I(z,x,t}_{i+1})|.$$ (2)

Then, the OCTA signals at each step y were averaged to improve flow contrast. Performing this process for all y values, OCTA volumetric data containing microvasculature flow information can be obtained. The *enface* angiograms are obtained by performing maximum intensity projection along the axial direction.

## 3 Results

### 3.1 OCT Structural Imaging Performance

**Fig. 4:**
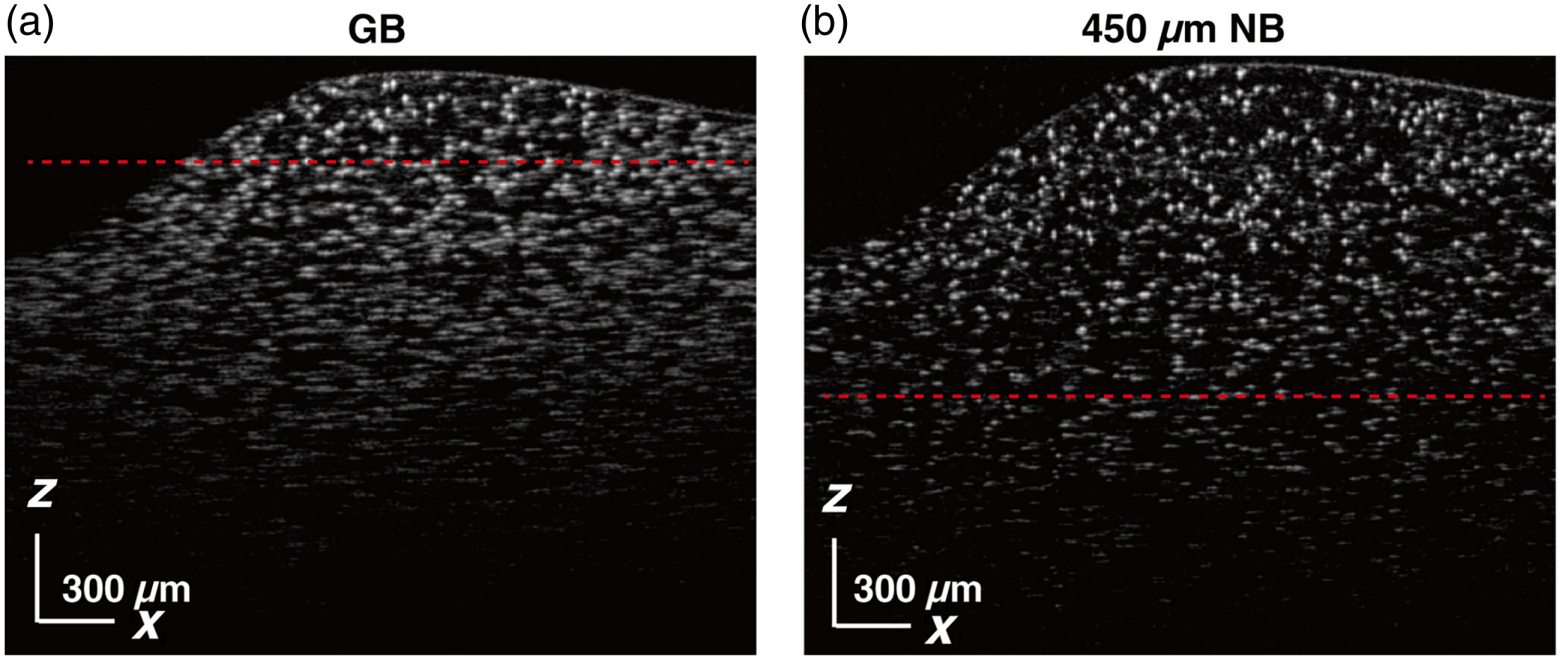
B-scan imaging results of 5  μm polystyrene microspheres with wearable probe using GB (a) and 450  μm NB (b). Scale bars at bottom left, 300  μm.

The GB and NB were tested with 5  μm polystyrene (PS) beads (XFJ100, XFnano) embedded in ultrasound gel (Ultrasonic Coupling, Cofoe). The phantom with beads concentration around $2\times 10^{5}/{\mathrm{mm}}^{3}$ was degassed with a centrifuge (10 min at 15,000 rpm, H1850, Cence). During the experiment, the probe is fixed on a bracket with the sample placed underneath. The integration time of the spectrometer was set to 20  μs, with 1024 A-scan in one B-scan. As shown in Fig. 4(a), the B-scan image acquired using the GB demonstrates recognizable beads only within a range of ∼160  μm near the focal plane, indicated by the red dashed line. This means that the GB maintains a lateral resolution above 8  μm only within this axial range, with areas farther from the focus displaying significant defocusing. By contrast, the NB provides a much wider range of distinguishable particles, maintaining a lateral resolution above 8  μm over a 450  μm axial range, achieving a 2.8-fold increase in DOF. As shown in Fig. 4(b), the NB allows for clear particle imaging across a larger depth range, with regions well above the red dashed line still displaying sharp and detailed features. This demonstrates that the NB can maintain high-resolution imaging over a significantly extended depth range.

**Fig. 5:**
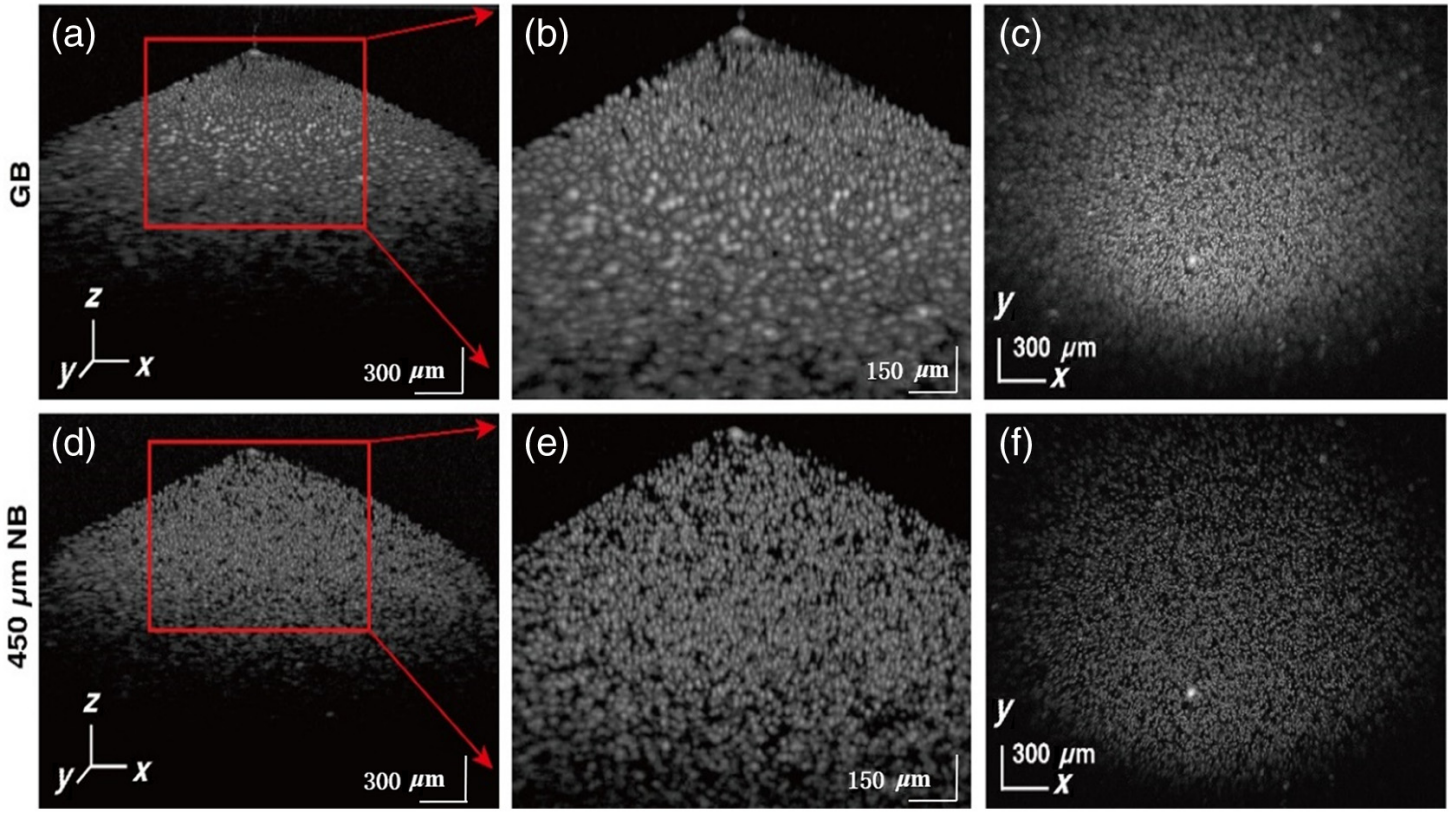
3D imaging results of 5  μm polystyrene microspheres with wearable probe using GB and 450  μm NB. (a) 3D imaging by GB. (d) 3D imaging by 450  μm NB, the red box in panels (a) and (d) is zoomed in as panels (b) and (e). (c) and (f) En face (x−y) images of the 3D volumetric data acquired by GB and 450  μm NB, respectively.

To better illustrate the differences between the two and the advantages of the NB, a 3D scan of the sample was performed with a lateral FOV of 3  mm×2.6  mm, with 1024 sampling points on the fast axis and 800 in the slow axis. The volumetric visualization of the 3D data is shown in Fig. 5. The imaging results of GB are shown in Figs. 5(a)–5(c). Figure 5(a) shows the overall effect, and the red box in it is zoomed in as Fig. 5(b), where the particles at the top are clearly imaged with high resolution, but the resolution rapidly decreases with depth, and the particles at the bottom are significantly widened due to beam defocus. By contrast, the imaging results using the 450  μm NB show no defocus phenomenon, as depicted in Figs. 5(d)–5(f). In Fig. 5(e), an enlarged view of the red box in Fig. 5(d), high-resolution imaging is maintained throughout the entire depth range, with particle sizes remaining consistent without the widening observed in Fig. 5(b). Figures 5(c) and 5(f) show the *enface* results of the volumetric data obtained using GB and NB, respectively, further highlighting the defocus phenomenon of the GB outside the DOF and the long focal depth advantage of the NB.

### 3.2 OCTA Imaging Performance

**Fig. 6:**
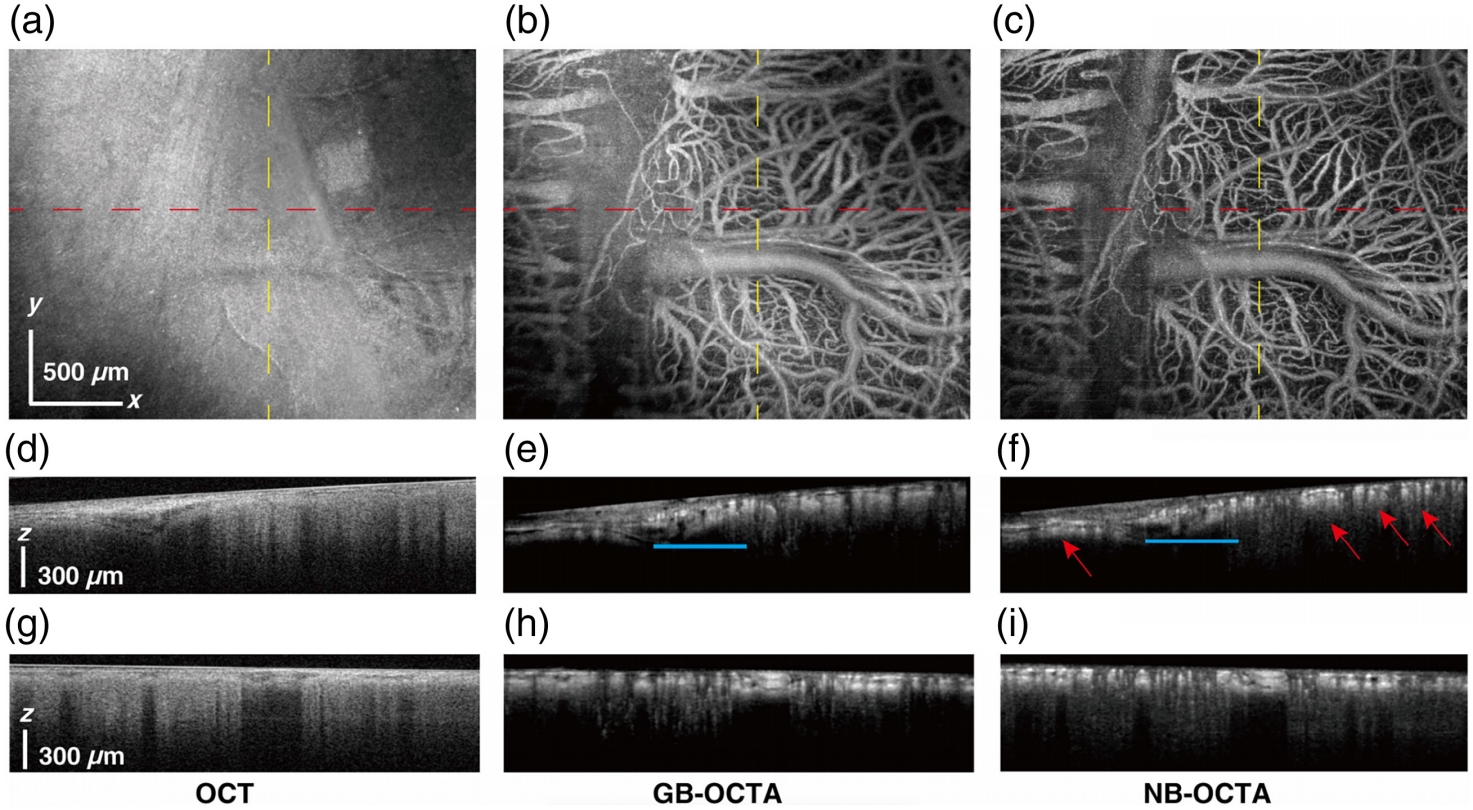
Imaging performance of the probe. (a) OCT image of the mouse brain, FOV, 2.5  mm×2  mm, scale bar at bottom left, 500  μm. (b) GB-OCTA image of the same region as panel (a). (c) NB-OCTA image of the same region as panel (a). Cross-sectional images of the red dash lines in panels (a)–(c) are shown in panels (d)–(f). Cross-sectional images of the yellow dash lines in panels (a)–(c) are shown in panels (g)–(i).

The connection between the probe and the mouse brain was described in our previous work.^18^ After wearing, the GB focus was placed at the axial position corresponding to the center of the imaging FOV. The imaging results are shown in Fig. 6. We took 800 B-scans in the slow scanner direction, and at each step, we repeated the B-scan five times; each of these B-scans consisted of 1024 A-lines in the fast-scanning direction, and the FOV was set to be 2.5  mm×2  mm. Figure 6(a) shows the *enface* result of the OCT data. Two positions were selected in the image (indicated by the red and yellow dashed lines), and corresponding OCT cross-sectional images are presented in Figs. 6(d) and 6(g). In Fig. 6(d), it can be observed that there is a height difference along the selected line. The height difference along this line is ∼200  μm. However, the maximum height difference was measured from the entire OCT 3D point cloud, representing the difference between the highest and lowest points across the entire OCT 3D surface, which was found to be 385  μm. The GB could only maintain a resolution better than 8  μm within an axial range of 160  μm, which is insufficient to cover the height difference of the sample surface; therefore, an obvious defocus phenomenon (the upper right corner position) can be observed in the GB-OCTA imaging result, shown in Fig. 6(b). After introducing the DOE, the wearable probe could maintain lateral resolution better than 8  μm in 450  μm, which exceeds the maximum height difference of the sample surface. Therefore, as shown in the NB-OCTA imaging result in Fig. 6(c), clear imaging could be achieved throughout the entire FOV without defocusing issues.

The advantages of NB-OCTA can also be observed from the comparison of the cross-sectional images. As shown in Figs. 6(e) and 6(f), the cross-sections represent the OCTA results at the positions corresponding to the red dashed lines in Figs. 6(b) and 6(c). The blood vessels are labeled by the blue lines in Figs. 6(e) and 6(f) are within the DOF of GB, thus achieving high resolution and signal-to-noise ratio (SNR) in both GB and NB imaging results. However, the blood vessels indicated by the red arrows in Fig. 6(f) are outside the GB focal range, thus only exhibiting high resolution and SNR in NB-OCTA. The clarity of the signal at the same position in Fig. 6(e) is significantly weaker than in Fig. 6(f). In addition, the contrast-to-noise ratio (CNR) and SNR were calculated using the signal intensity and background intensity across the entire enface image, rather than selecting a specific region. Higher CNR helps to distinguish blood vessels from surrounding tissue, whereas higher SNR improves image clarity and visibility of details, aiding in the evaluation of vascular connectivity. In Fig. 6(b), CNR=20.57  dB and SNR=22.84  dB; in Fig. 6(c), CNR=21.95  dB and SNR=25.81  dB, both demonstrating better performance of the NB.

$${\mathrm{CNR}}_{\text{enface OCTA}}=\frac{\overset{¯}{I(x,y){|}_{M(x,y)=1}}-\overset{¯}{I(x,y){|}_{\mathrm{Bck}}}}{\sigma_{I(x,y)|\mathrm{Bck}}},$$ (3)

$${\mathrm{SNR}}_{\text{enface OCTA}}=\frac{\overset{¯}{I(x,y){|}_{M(x,y)=1}}}{\sigma_{I(x,y)|\mathrm{Bck}}}.$$ (4)

The GB-OCTA and NB-OCTA enface images were first normalized. A threshold was then applied to generate binary images to differentiate blood vessels from the background. The threshold was selected based on the grayscale pixel values of the enface images and empirical observation. Regions corresponding to blood vessels, with higher intensity than the threshold, were assigned a value of 1, whereas regions corresponding to the background, with lower intensity than the threshold, were assigned a value of 0. This binary vessel map was represented as a skeleton map (M). $\overset{¯}{I(x,y){|}_{M(x,y)=1}}$ is the mean value of the intensities of the pixels that corresponded to the skeleton map in the *enface* OCTA image, $\overset{¯}{I(x,y){|}_{\mathrm{Bck}}}$ represents the average intensity of pixels in the background (tissue) area of the *enface* image, and $\sigma_{I(x,y)|\mathrm{Bck}}$ is the standard deviation of the background signals.

**Fig. 7:**
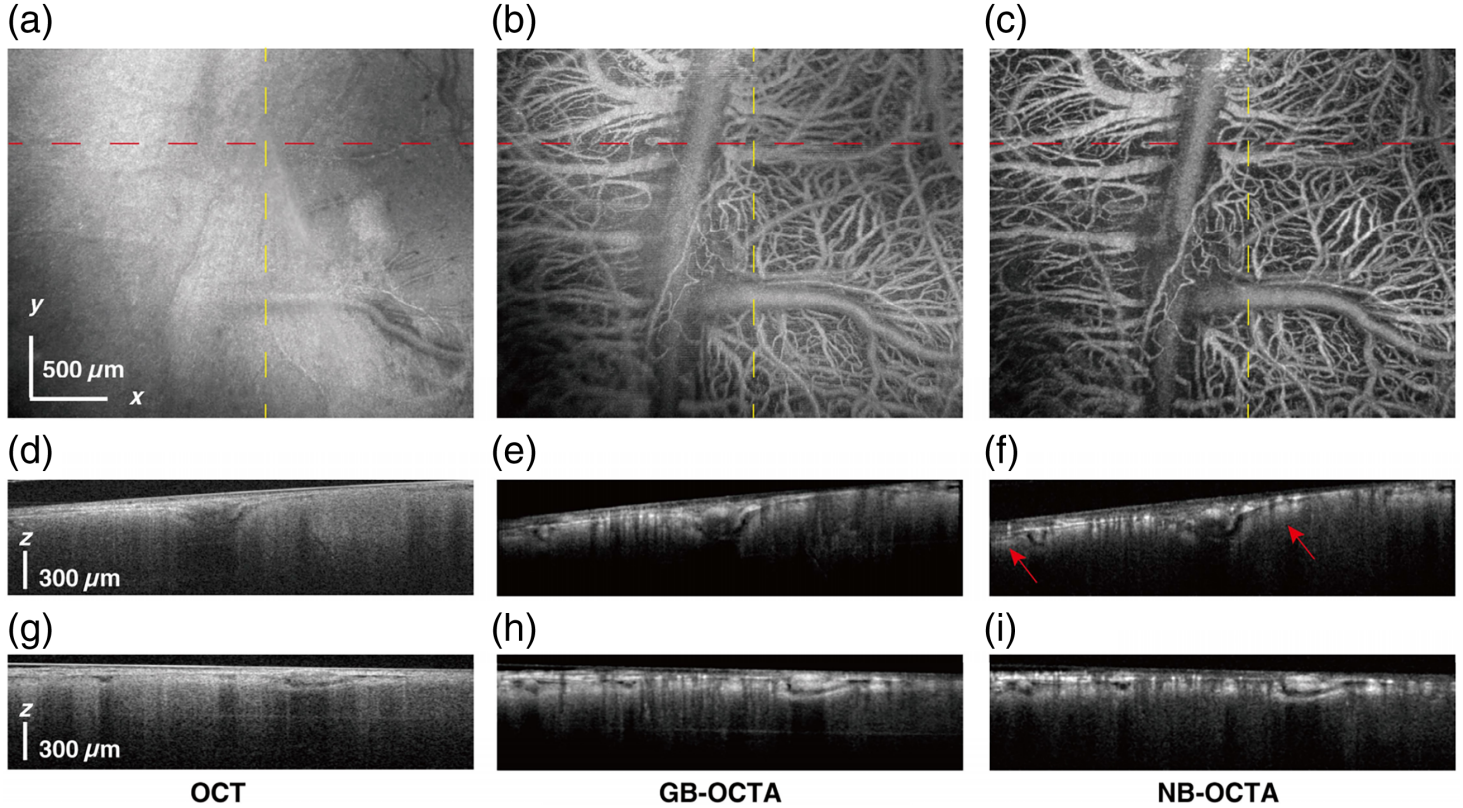
Imaging performance of the probe. (a) OCT image of the mouse brain, FOV, 3  mm×2.4  mm, scale bar at bottom left, 500  μm. (b) GB-OCTA image of the same region as panel (a). (c) NB-OCTA image of the same region as panel (a). Cross-sectional images of the red dash lines in panels (a)–(c) are shown in panels (d)–(f). Cross-sectional images of the yellow dash lines in panels (a)–(c) are shown in panels (g)–(i).

In the experiment described above, the FOV was 2.5  mm×2  mm. This time, the orientation of the wearable probe was kept unchanged, the FOV was expanded by 20% to image a region of 3  mm×2.4  mm, and the acquisition scheme remained the same at 1024×800×5. Due to the enlargement of the imaging field, the maximum height difference increased from 385 to 425  μm, and as a result, the GB-OCTA imaging shown in Fig. 7(b) exhibits more pronounced defocusing (as shown in the upper right corner of the image), whereas the 450  μm DOF of the NB can still cover the maximum height difference of 425  μm. Therefore, the imaging result of NB-OCTA in Fig. 7(c) can still maintain high-resolution imaging without defocusing throughout the whole FOV.

Similarly, the comparison of OCTA cross-sectional images shown in Figs. 7(e) and 7(f) also highlights the advantages of NB-OCTA, and the blood vessel labeled by the red arrow in Fig. 7(f) exhibits higher resolution and SNR in NB-OCTA. In Fig. 7(b), CNR=15.49  dB and SNR=19.77  dB; in Fig. 7(c), CNR=16.37  dB and SNR=22.07  dB, demonstrating better performance of the NB in the wearable probe.

**Fig. 8:**
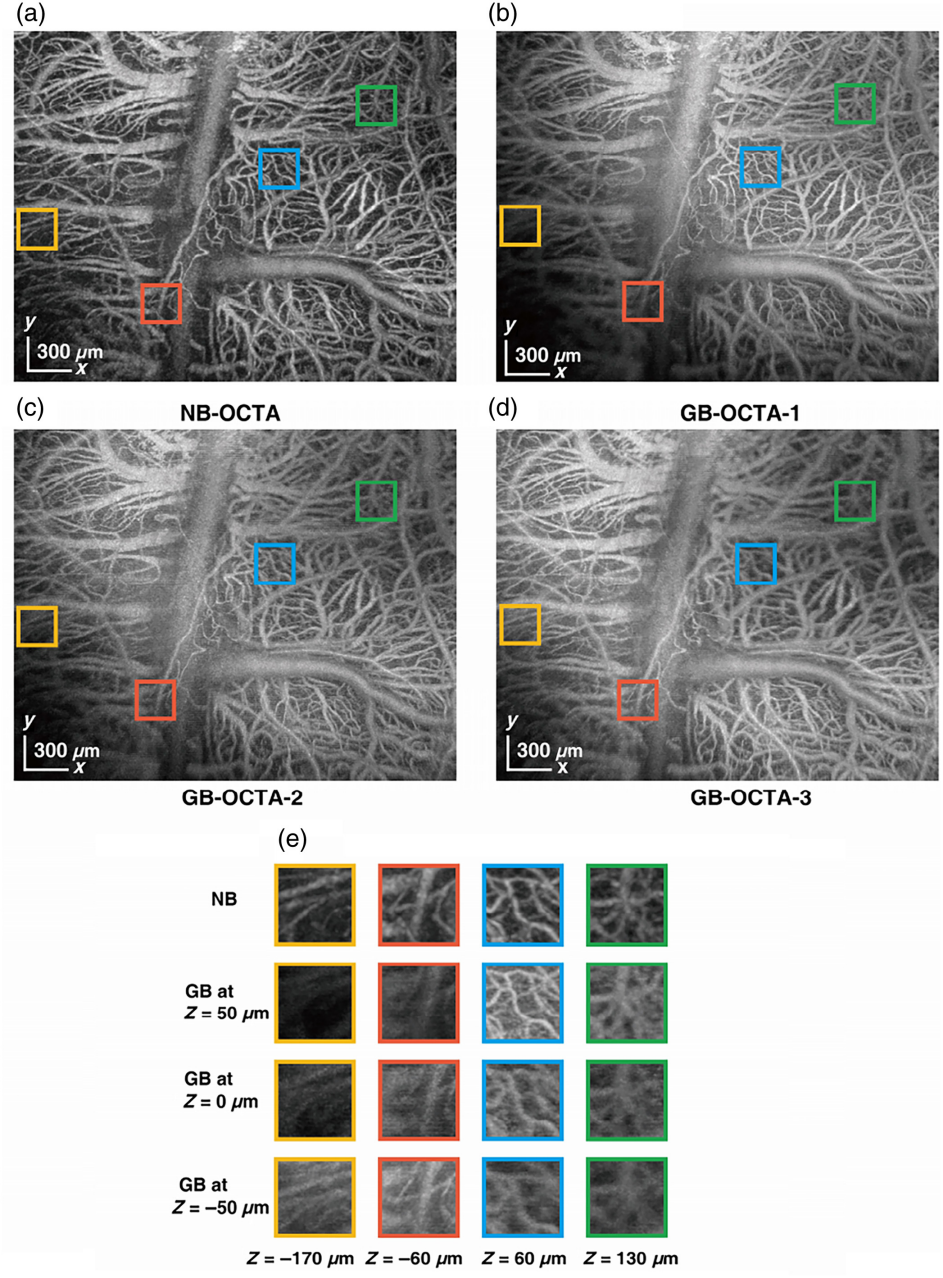
OCTA imaging of mouse brain (3  mm×2.4  mm) with wearable probe using GB and 450  μm NB. (a) *Enface* (x−y) image acquired by 450  μm NB, scale bars at bottom left, 300  μm. (b) *Enface* (x−y) image acquired by GB with the focal position at z=50  μm. (c) *Enface* (x−y) image acquired by GB with the focal position at z=0  μm. (d) *Enface* (x−y) image acquired by GB with the focal position at z=−50  μm. The yellow, red, blue, and green boxes in panels (a–d) are zoomed in panel (e), indicating axial positions of −170  μm, −60  μm, 60  μm, and 130  μm, respectively.

To further demonstrate the advantage of the NB in the wearable probe, the same FOV of Fig. 7 is used, and the position of the beam focus is adjusted by changing the number of spacers in the probe. This experiment focuses the GB at three different axial positions (z=50  μm, z=0  μm, z=−50  μm), as shown in Figs. 8(b)–8(d). The *enface* image acquired with NB is shown in Fig. 8(a). The yellow, red, blue, and green boxes in Figs. 8(a)–8(d) are zoomed in Fig. 8(e), indicating axial positions of −170  μm, −60  μm, 60  μm, and 130  μm, respectively, for 450  μm NB-OCTA, high resolution is maintained at all four positions, whereas for GB-OCTA, no matter where the focus is, the DOF of the beam cannot cover the height difference of the sample surface (425  μm), so there will always be positions out of focus. This experiment provides a more comprehensive explanation of the necessity of using NB in the wearable probe.

## 4 Conclusion and Discussion

This work is a continuation of our previous work, aiming to address the issue of insufficient DOF in wearable OCTA probes when imaging the mouse brain due to the height difference of the sample. By introducing the NB into the wearable probe, it overcomes the trade-off between lateral resolution and DOF, achieving a lateral resolution better than 8  μm within an axial space of 450  μm.

The OCT imaging of PS bead samples demonstrated the significant defocusing phenomenon of GB outside the DOF and the advantage of the NB. In the experiments of mouse brain imaging, the OCTA imaging results using GB and NB were compared. The introduction of the NB enabled the wearable probe to overcome the surface height difference of the sample and maintain high-resolution imaging without defocusing. The wearable OCTA probe is not only capable of imaging the awake mouse brain but also capable of performing fast, high-resolution, non-stitched imaging on the curved mouse brain in a wearable manner.

This work contributes to the advancement of OCTA applications in the brain, but there are still areas that require further improvement. For example, the light wavelength used in this paper is relatively short, resulting in a shallow penetration depth, which necessitates craniotomy for measurements on mice. In the future, longer wavelength systems could be utilized, or combined with adaptive optics technology capable of wavefront shaping to increase the penetration depth,^30^ allowing for non-craniotomy OCTA and wearable OCTA research on mice. The wearable probe used in this paper is a first-generation design, with room for optimization in terms of weight, size, and optical path structure. For example, a lightweight fiber collimator could be constructed, lighter materials could be used for the overall framework, and unnecessary solid parts in the structure could be optimized. The wearable probe used in this paper employs a 12.7 mm diameter double-cemented achromatic lens, which can produce aberrations during large FOV scanning. In the future, a small aperture flat-field scanning lens could be designed to produce a uniform spot size at all scanning positions on the image plane, achieving higher quality large FOV imaging results.

In future research, other technologies and algorithms can be integrated into wearable NB-OCTA, such as blood flow velocity measurement and oxygen saturation measurement, providing more biochemical monitoring information alongside angiography. We believe that this work holds promising prospects for future applications and will promote our understanding of brain health and neurological disorders.

## Data Availability

The datasets generated and/or analyzed during the current study are available from the corresponding author on reasonable request.

## References

[1] Spaide R. F. et al., “Optical coherence tomography angiography,” Prog. Retin. Eye Res. 64, 1–55 (2018). PRTRES 1350-9462 10.1016/j.preteyeres.2017.11.003 29229445 PMC6404988

[r2] Lin L. Wang L. V., “The emerging role of photoacoustic imaging in clinical oncology,” Nat. Rev. Clin. Oncol. 19, 365–384 (2022). 10.1038/s41571-022-00615-3 35322236

[3] Regnault G. et al., “Spatial resolution in optical coherence elastography of bounded media,” Biomed. Opt. Express 13(9), 4851–4869 (2022). BOEICL 2156-7085 10.1364/BOE.469019 36187272 PMC9484430

[4] Silasi G. et al., “Intact skull chronic windows for mesoscopic wide-field imaging in awake mice,” J. Neurosci. Methods 267, 141–149 (2016). JNMEDT 0165-0270 10.1016/j.jneumeth.2016.04.012 27102043 PMC5075450

[5] Bergel A. et al., “Adaptive modulation of brain hemodynamics across stereotyped running episodes,” Nat. Commun. 11, 6193 (2020). NCAOBW 2041-1723 10.1038/s41467-020-19948-7 33273463 PMC7713412

[6] Cao V. Y. et al., “In vivo two-photon imaging of experience-dependent molecular changes in cortical neurons,” J. Vis. Exp. 71, e50148 (2013). 10.3791/50148 PMC3582675 23329071

[7] Nasiriavanaki M. et al., “High-resolution photoacoustic tomography of resting-state functional connectivity in the mouse brain,” Proc. Natl. Acad. Sci. U. S. A. 111(1), 21–26 (2014). 10.1073/pnas.1311868111 24367107 PMC3890828

[8] Li Y. et al., “Aging-associated changes in cerebral vasculature and blood flow as determined by quantitative optical coherence tomography angiography,” Neurobiol. Aging 70, 148–159 (2018). NEAGDO 0197-4580 10.1016/j.neurobiolaging.2018.06.017 30007164 PMC6119107

[9] Slupe A. M. Kirsch J. R., “Effects of anesthesia on cerebral blood flow, metabolism, and neuroprotection,” J. Cereb. Blood Flow Metab. 38(12), 2192–2208 (2018). 10.1177/0271678X18789273 30009645 PMC6282215

[10] Dombeck D. A. et al., “Imaging large-scale neural activity with cellular resolution in awake, mobile mice,” Neuron 56(1), 43–57 (2007). NERNET 0896-6273 10.1016/j.neuron.2007.08.003 17920014 PMC2268027

[11] Li Y. et al., “Procedure and protocols for optical imaging of cerebral blood flow and hemodynamics in awake mice,” Biomed. Opt. Express 11(6), 3288–3300 (2020). BOEICL 2156-7085 10.1364/BOE.394649 32637255 PMC7316002

[12] Rakymzhan A. et al., “Differences in cerebral blood vasculature and flow in awake and anesthetized mouse cortex revealed by quantitative optical coherence tomography angiography,” J. Neurosci. Methods 353, 109094 (2021). JNMEDT 0165-0270 10.1016/j.jneumeth.2021.109094 33549637 PMC7990717

[13] Feng G. et al., “High-resolution structural and functional retinal imaging in the awake behaving mouse,” Commun. Biol. 6, 572 (2023). 10.1038/s42003-023-04896-x 37248385 PMC10227058

[14] Guo H. et al., “Detachable head-mounted photoacoustic microscope in freely moving mice,” Opt. Lett. 46(24), 6055–6058 (2021). OPLEDP 0146-9592 10.1364/OL.444226 34913906

[15] Ozbay B. N. et al., “Three dimensional two-photon brain imaging in freely moving mice using a miniature fiber-coupled microscope with active axial scanning,” Sci. Rep. 8, 8108 (2018). SRCEC3 2045-2322 10.1038/s41598-018-26326-3 29802371 PMC5970169

[16] Miao P. et al., “Laser speckle contrast imaging of cerebral blood flow in freely moving animals,” J. Biomed. Opt. 16(9), 090502 (2011). JBOPFO 1083-3668 10.1117/1.3625231 21950906

[17] Chen C.-L. Wang R. K., “Optical coherence tomography-based angiography,” Biomed. Opt. Express 8(2), 1056–1082 (2017). BOEICL 2156-7085 10.1364/BOE.8.001056 28271003 PMC5330554

[18] Guo X. et al., “Wearable optical coherence tomography angiography probe for freely moving mice,” Biomed. Opt. Express 14(12), 6509–6520 (2023). BOEICL 2156-7085 10.1364/BOE.506513 38420312 PMC10898568

[19] Drexler W. Fujimoto J. G., “Optical coherence tomography: technology and applications,” in Conf. Lasers & Electro-Opt. Eur. Int. Quantum Electron. Conf. (CLEO EUROPE/IQEC), Munich, Germany, p. 1 (2013).

[20] Wang J. et al., “Invariant features-based automated registration and montage for wide-field OCT angiography,” Biomed. Opt. Express 10(1), 120–136 (2019). BOEICL 2156-7085 10.1364/BOE.10.000120 30775088 PMC6363196

[r21] Zhang Q. et al., “Wide-field optical coherence tomography-based microangiography for retinal imaging,” Sci. Rep. 6, 22017 (2016). SRCEC3 2045-2322 10.1038/srep22017 26912261 PMC4766473

[22] He B. et al., “Whole-brain micro-vascular imaging using robot-assisted optical coherence tomography angiography,” IEEE J. Sel. Top. Quantum Electron. 29(4), 1–9 (2022). IJSQEN 1077-260X 10.1109/JSTQE.2021.3054892

[23] Zhao J. et al., “Flexible method for generating needle-shaped beams and its application in optical coherence tomography,” Optica 9(8), 859–867 (2022). 10.1364/OPTICA.456894 37283722 PMC10243785

[24] Guo X. et al., “Visualizing cortical blood perfusion after photothrombotic stroke in vivo by needle-shaped beam optical coherence tomography angiography,” PhotoniX 5, 7 (2024). 10.1186/s43074-024-00124-9

[25] Cao R. et al., “Optical-resolution photoacoustic microscopy with a needle-shaped beam,” Nat. Photonics 17, 89–95 (2023). NPAHBY 1749-4885 10.1038/s41566-022-01112-w 38149029 PMC10751030

[26] Lorenser D. et al., “Energy-efficient low-Fresnel-number Bessel beams and their application in optical coherence tomography,” Opt. Lett. 39(3), 548–551 (2014). OPLEDP 0146-9592 10.1364/OL.39.000548 24487862

[r27] Leitgeb R. et al., “Extended focus depth for Fourier domain optical coherence microscopy,” Opt. Lett. 31(16), 2450–2452 (2006). OPLEDP 0146-9592 10.1364/OL.31.002450 16880852

[28] Lee K.-S. Rolland J. P., “Bessel beam spectral-domain high-resolution optical coherence tomography with micro-optic axicon providing extended focusing range,” Opt. Lett. 33(15), 1696–1698 (2008). OPLEDP 0146-9592 10.1364/OL.33.001696 18670507

[29] Chen T.-W. et al., “Ultrasensitive fluorescent proteins for imaging neuronal activity,” Nature 499, 295–300 (2013). 10.1038/nature12354 23868258 PMC3777791

[30] Pircher M. Zawadzki R. J., “Review of adaptive optics OCT (AO-OCT): principles and applications for retinal imaging,” Biomed. Opt. Express 8(5), 2536–2562 (2017). BOEICL 2156-7085 10.1364/BOE.8.002536 28663890 PMC5480497

